# BMF-AS1/BMF Promotes Diabetic Vascular Calcification and Aging both *In Vitro* and *In Vivo*

**DOI:** 10.14336/AD.2022.0427

**Published:** 2023-02-01

**Authors:** Xiao Lin, Qun-Yan Xiang, Shuang Li, Wan-Ling Song, Yan-Jiao Wang, Yu-Qing Ni, Yan Zhao, Chen Li, Yi Wang, Hua-Hua Li, Zhen Liang, Jun-Kun Zhan, You-Shuo Liu

**Affiliations:** ^1^Department of Geriatrics, the Second Xiangya Hospital of Central South University, Hunan, China.; ^2^Institute of Aging and Age-related Disease Research, Central South University, Hunan, China.; ^3^Department of Radiology, the Second Xiangya Hospital of Central South University, Hunan, China.; ^4^Department of Biochemistry, University of Oxford, Oxford, UK.; ^5^Department of Geriatrics, Hunan Provincial People's Hospital, the First Affiliated Hospital of Hunan Normal University, Hunan, China.; ^6^Department of Geriatrics, Shenzhen People's Hospital (the Second Clinical Medical College, Jinan University; the First Affiliated Hospital, Southern University of Science and Technology), Guangdong, China

**Keywords:** BMF-AS1, BMF, vascular calcification and aging, vascular smooth muscle cells, diabetes mellitus, coronary calcification score

## Abstract

Vascular calcification and aging often increase morbidity and mortality in patients with diabetes mellitus (DM); however, the underlying mechanisms are still unknown. In the present study, we found that Bcl-2 modifying factor (BMF) and BMF antisense RNA 1 (BMF-AS1) were significantly increased in high glucose-induced calcified and senescent vascular smooth muscle cells (VSMCs) as well as artery tissues from diabetic mice. Inhibition of BMF-AS1 and BMF reduced the calcification and senescence of VSMCs, whereas overexpression of BMF-AS1 and BMF generates the opposite results. Mechanistic analysis showed that BMF-AS1 interacted with BMF directly and up-regulated BMF at both mRNA and protein levels, but BMF did not affect the expression of BMF-AS1. Moreover, knocking down BMF-AS1 and BMF suppressed the calcification and senescence of VSMCs, and BMF knockout (BMF^-/-^) diabetic mice presented less vascular calcification and aging compared with wild type diabetic mice. In addition, higher coronary artery calcification scores (CACs) and increased plasma BMF concentration were found in patients with DM, and there was a positive correlation between CACs and plasma BMF concentration. Thus, BMF-AS1/BMF plays a key role in promoting high glucose-induced vascular calcification and aging both *in vitro* and *in vivo*. BMF-AS1 and BMF represent potential therapeutic targets in diabetic vascular calcification and aging.

Diabetes mellitus (DM) is a serious metabolic disease, which severely impairs the lives and health of individuals, families, and societies all over the world. The global prevalence of DM was estimated to be 9.3% (463 million people) in 2019 and is predicted to rise to 10.2% (578 million) and 10.9% (700 million) by 2030 and 2045, respectively [1]. Patients with DM often have vascular calcification, which is characterized by abnormal calcium and phosphate salt deposition in the vascular wall, and is commonly observed in older patients, and patients with DM and chronic kidney disease [2, 3]. Vascular calcification is a specific phenotype of vascular aging and mainly involves the vascular smooth muscle cells (VSMCs) of the medial layer of the artery [4]. Although several molecules have been shown to participate in the pathogenesis of vascular calcification and aging [5, 6, 7], it is likely that more are yet to be discovered.

Long noncoding RNAs (lncRNAs) are a class of transcript longer than 200 bases with no protein coding ability [6]. Antisense lncRNAs are a subclass of lncRNAs that are transcribed in an antisense orientation with respect to the protein coding gene [8, 9]. Recent evidence has verified that lncRNAs play a key role in regulating the pathophysiology of different human diseases, including vascular calcification and aging [2, 7]. For example, the lncRNA Dnm3os enhances promoter H3K9ac, leading to chromatin relaxation, upregulation of inflammatory targets and macrophage dysfunction in DM [10]. However, there are functional lncRNAs involved in vascular calcification and aging yet to be identified.

Bcl-2 modifying factor (BMF), a pro-apoptotic gene, plays a crucial role in regulating different cellular functions under hyperglycemia [11, 12] and promotes apoptosis in renal proximal tubular cells in diabetic mice [11]. Corsten et al. reported that BMF also promotes VSMC apoptosis by regulating miR-221/222 expression [12]. Our previous study demonstrated for the first time that BMF is involved in regulating VSMC calcification and senescence under the condition of high glucose [6]. However, the role of BMF modulation of diabetic vascular calcification and aging *in vivo* and the specific mechanism needs to be further explored.

In the present study, we evaluated if BMF antisense RNA 1 (BMF-AS1) could interact with BMF and regulate its expression, and whether they could promote VSMC calcification and senescence *in vitro*. Besides, we explored the role of BMF on diabetic vascular calcification and aging *in vivo* using a BMF knock out (BMF^-/-^) mice model. Moreover, we tested the plasma concentration of BMF in patients with DM and analyzed the correlation between plasma BMF concentration and coronary artery calcification score (CACs).

## MATERIALS AND METHODS

### Cell culture

Human aorta VSMCs (HA-VSMCs) were purchased from ATCC (ATCC-CRL-1999) and cultured in Dulbecco’s Modified Eagle’s Medium (DMEM) supplemented with 10% fetal bovine serum (FBS), streptomycin (100 μg/mL) and penicillin (100 U/mL). HA-VSMCs were incubated at different concentrations of glucose (5, 10, 20, 30 mM) or for various times in the presence of either a normal concentration of glucose (NG, 5 mM) or a high concentration (HG, 30 mM). A 30 mM mannitol condition was used as a hyperosmotic control (HO). The culture medium was refreshed every 2 days and cells were cultured at 37 °C with a humidified atmosphere of 5% CO_2_.

### Cell transfection

For cell transfection, siRNA-BMF-AS1-84, siRNA-BMF-AS1-140 and siRNA-BMF-AS1-312 were designed and synthesized by GenePharma Co. Ltd (Shanghai, China). siRNA-BMF-AS1 (50 nM) oligos and BMF small interfering RNA (siBMF) oligos (50 nM) (Qiagen, Germany) were transfected into VSMCs using lipofectamine 3000 (L3000001, Invitrogen, Waltham, MA, USA) according to the manufacturer’s instructions. Then, the cells were cultured in DMEM without FBS for 6 h and then for another 48 h with 10% FBS. Finally, the efficiency of the siRNAs was measured by quantitative reverse transcription-polymerase chain reaction (qRT-PCR) and the most effective siRNA-BMF-AS1 was used for the downstream functional experiments. The sequences of siRNA-BMF-AS1 and siBMF used in this study are shown in the Supplementary Table. 1.

For the overexpression experiments, a BMF or BMF-AS1 overexpression sequence was inserted into a plasmid vector. Then, 5 µg/ml of plasmid was transfected into VSMCs using lipofectamine 3000 according to the manufacturer’s instructions. After that, the cells were cultured in DMEM without FBS for 6 h and then for another 96 h with 10% FBS. The number of transfected cells was observed under a fluorescence microscope when transfection rate was highest. VSMCs transfected for 72 h achieved the highest transfection ratio of more than 60% and were used for the downstream functional experiments. The transfection efficiency is shown in Supplementary Fig. 1.

### Alizarin Red S staining and Von Kossa staining

The calcification of VSMCs and artery tissue sections was detected using Alizarin Red S and Von Kossa staining in a similar way to that described previously [13]. For Alizarin Red S staining, VSMCs were cultured in different conditions for 18 days and then fixed in 4% paraformaldehyde for 30 min at room temperature (RT). The groups were divided as follows: VSMCs + 5 mM β-glycerophosphate (β-GP) + 5 mM glucose (NG); VSMCs + 5 mM β-GP + 30 mM mannitol (HO); and VSMCs + 5 mM β-GP + 30 mM glucose (HG). Artery tissues were paraffin-embedded, deparaffinized and rehydrated. Finally, VSMCs or artery tissue sections were stained with 1% Alizarin Red S (pH 4.2, Sigma) for 5 min at 37 °C.

For Von Kossa staining, artery tissue sections were incubated with 5% silver nitrate (Sigma) for 1 h under ultraviolet light, followed by incubation in sodium thiosulfate for 10 min. The stained matrix was assessed and photographed using a digital microscope. For the quantification of calcium levels, cells and arteries were washed with phosphate-buffered saline (PBS) and decalcified with 0.6 N HCl for 24 h; the calcium content in the HCl-dissolved supernatants was then analyzed using the o-cresolphthalein method. Total protein was detected using the Bradford protein assay. The calcium content was normalized to protein content and presented as μg of calcium/mg of protein.

### Senescence-associated β-galactosidase (SA-β-gal) staining

SA-β-gal staining was performed using a senescence-associated β-galactosidase staining kit (Cat#: C0602, Beyotime Institute of Biotechnology, Shanghai, China) according to the manufacturer’s instructions. Briefly, VSMCs cultured under different conditions were fixed in β-galactosidase fixation solution (2% formaldehyde/0.2% glutaraldehyde in PBS) for 15 min and then washed with PBS. Frozen sections were made from artery tissues and then fixed in β-galactosidase fixation solution. The cells or frozen artery tissue sections were stained in SA-β-gal staining solution (pH 6.0) for at least 16 h at 37 °C. Ten random fields were imaged per section. The percentage of senescent cells or the areas of aging arteries was determined using Image-Pro Plus software (version 6.0).

### qRT-PCR

Total RNA was extracted from cultured VSMCs and mice artery tissues using TRIzol Reagent (Invitrogen, Cat#: 15596-026) [3]. cDNA was synthesized from 1 μg of total RNA using RevertAid™ H Minus First Strand cDNA Synthesis Kit (Fermentas, Cat#: K1631) and qRT-PCR was carried out to analyze the RNA levels of BMF and BMF-AS1. Briefly, 25 μl of reactants were incubated in a 96-well optical plate at 95 °C for 5 min, followed by 40 cycles of 95 °C for 20 seconds, 60 °C for 20 seconds and 72 °C for 20 seconds. The relative RNA level was calculated using the comparative threshold cycle (2^-ΔΔCT^) method and normalized to β-actin value within the sample. The primers used in this study are shown in the Supplementary Table. 2.

### Immunofluorescence (IF) assay

The location of the BMF protein in VSMCs was examined by IF. Briefly, VSMCs were fixed with 4% paraformaldehyde for 30 min at RT and blocked with 5% fetal calf serum for 1 h, followed by incubation with anti-BMF (Cat#: ab9655, Abcam; 1:100,) overnight at 4 °C. The next day, cells were incubated with Alexa Fluor® 555 goat anti-rabbit IgG (Cat#: A21428a, Invitrogen, 1:100) for 30 min at RT. The nucleus was stained with 4, 6-diamidino-2-phenylindole (DAPI, Molecular Probe, Cat#: D1306). The results were observed under a Leica TCS SP5 laser confocal scanning microscope (Mannheim, Germany) and analyzed using Image-Pro Plus software (version 6.0).

### Fluorescent in situ hybridization (FISH)

FISH was performed to detect the location of BMF-AS1 using a Ribo™ Fluorescent I*n Situ* Hybridization Kit (Cat#: C10910, RiboBio Co., Ltd. Guangzhou, China). VSMCs were fixed with 4% paraformaldehyde for 30 min at RT and then incubated with 200 μl pre-hybridization buffer for 30 min at 37 °C. BMF-AS1 FISH probe mix or U6 was mixed with pre-hybridization buffer and then incubated with VSMCs at 37 °C overnight. The nuclei were stained with DAPI (Molecular Probe, Cat#: D1306). The results were observed under a Leica TCS SP5 laser confocal scanning microscope and analyzed using Image-Pro Plus software (version 6.0).

### Western blot (WB) analysis

WB was performed as previously described [6]. Briefly, proteins were extracted from VSMCs or mouse artery tissues and quantified using a BCA Protein kit (Cat#: P0010S, Beyotime Biotechnology, Shanghai, China). Samples containing 30 µg of protein were loaded onto SDS-PAGE and then transferred to a polyvinylidene fluoride (PVDF) membrane (Millipore, Billerica, USA). After blocking with 5% non-fat milk, the membrane was incubated with primary antibodies overnight at 4 °C. The primary antibodies used were anti-Runx2 antibody (Cat#: ab76956, Abcam, 1:1000), anti-ALP antibody (Cat#: GTX119505, Genetex, 1:500), anti-p21 antibody (Cat#: 2947, CST, 1:1000), anti-p16 antibody (Cat#: 10883-1-AP, Proteintech, 1:500), anti-BMF antibody (Cat#: 18298-1-AP, Proteintech, 1:500) and anti-β-actin antibody Cat#: (Cat#: 66009-1-Ig, Proteintech, 1:2000). The next day, the membranes were washed with PBS and then incubated with an appropriate secondary antibody (Cat#: SA00001-1 or SA00001-2, Proteintech, 1:5000) in 2% non-fat milk for 1 h. The immunoreactive bands were detected using a chemiluminescence kit (Cat#: RPN2232, Amersham Biosciences Ltd., UK) and then analyzed using Image-Pro Plus software (version 6.0). The relative protein expression level was normalized to the intensity of the β-actin band.

### RNA pull-down

RNA pull-down assays were carried out using a Pierce™ Magnetic RNA-Protein Pull-Down Kit (Cat#: 20164, Thermo Fisher Scientific). Briefly, for each assay, biotinylated BMF-AS1 was conjugated to streptavidin magnetic beads. Then, the conjugated beads were incubated with the lysates from VSMCs in binding reaction buffer at 4 °C for 60 min with rotation. Next, the bound RNA-protein complexes were washed and eluted from the magnetic beads. Finally, RNAs in the complexes were purified and the enrichment patterns of BMF mRNA and protein were measured using qRT-PCR and WB, respectively.

### RNase protection assay (RPA)

Cultured VSMCs were transfected with siRNA-BMF-AS1, and total RNA was extracted. RNase digestive solution was then added. The cells were kept at 37 °C for 30 min. Then, 10 μl of 10% SDS mix containing 20 μl of protease K (10 μg/μl) was added and kept at 37 °C for 10 min to terminate digestion. Finally, the level of BMF mRNA was detected by qPCR to determine the protective effect of BMF-AS1 on BMF mRNA.

### mRNA stability assay

VSMCs were treated with 5 μg/ml of actinomycin D following the manufacturer’s recommendations after transfecting with siRNA-BMF-AS1 for 16 h. The total RNA was collected and the level of BMF mRNA was measured using qPCR at 0, 2, 4, 6 and 8 h, respectively. For this measurement, 18s RNA was used as the reference control because it is very stable and minimally affected by nucleases. The primer sequence of 18s was as follows: Forward:5’-CAGCCACCCGAGATTGAGCA-3’ Reverse: 5’-TAGTAGCGACGGGCGGTGTG-3’

### Animal study

The animal investigation conformed to published guidelines (National Research Council, The Care and Use of Laboratory Animals, 7^th^ ed. Washington, DC: National Academies Press; 1996). All animal studies were formally approved by the Ethics Committee of the Second Xiangya Hospital, Central South University (Cat#: 2018015). Six- to eight-week-old C57BL/6-Bmf^em1Smoc^ (Cat#: NM-KO-190003) mice (three males and three females) were purchased from Shanghai Model Organisms Center, Inc. (Shanghai, China). The heterozygote Bmf knockout mice (Bmf^-/+^) were crossbred with each other to obtain homozygous Bmf knockout mice (Bmf^-/-^). Genotype was determined from mouse tail biopsies lysed in mouse tail lysate (300 μL) and proteinase K (6 μL) in a water bath overnight at 55 °C. The next day, DNA was extracted for PCR amplification. Finally, agarose gel electrophoresis was performed to detect the amplified products. The identifying primer sequence is given in Supplementary Tab. 3 and representative results of genotyping are shown in Supplementary Fig. 2A and B.

All mice were housed with 12 h daylight/darkness in the animal house of the Second Xiangya Hospital. To generate Bmf^-/-^ diabetic mice, streptozotocin (STZ; 50 mg/kg; Sigma) was freshly dissolved in 0.1 M phosphate-citrate buffer (pH 4.5) and injected into mice on five consecutive days. The non-DM control mice received the same dose of citrate buffer. Blood glucose levels were monitored after 3 days, and levels > 16.67 mM indicated the onset of DM [14-16]. The body weight and the level of blood glucose were taken before and every week after STZ injection. The pathological characteristics are shown in Supplementary Fig. 2C and D. After 12 weeks, mice were sacrificed, and the thoracic aortas were dissected. The expression of BMF and BMF-AS1 mRNA and protein were measured by qRT-PCR and WB, respectively, and immunohistochemistry was carried out to test the levels of BMF, Runx2 and p21 protein in aortic tissues. Alizarin Red S and Von Kossa staining were used to detect artery calcification. SA-β-gal staining was carried out to detect artery aging. Total protein was quantified using the Bradford protein assay.

### Immunohistochemistry analysis

The expression of BMF, Runx2 and p21 in the aorta tissues were examined by immunohistochemistry as described previously [13]. Briefly, the artery tissue sections were baked at 65 °C for 2 h, followed by two rounds of dewaxing in xylene for 10 min and dehydrating in 99%, 95% and 75% ethanol for 5 min each. Sections were then incubated with hydrogen peroxide at RT in a dark box for 10 min to diminish endogenous peroxidases, and trypsin-EDTA solution was used for antigen retrieval. Next, sections were blocked using 5% fetal serum for 1 h and incubated with a specific primary antibody, including anti-BMF antibody (Cat#: 17A9, ENZO Life Sciences, 1:200), anti-Runx2 antibody (Cat#: ab76956, Abcam, 1:500) and anti-p21 antibody (Cat#: 2947, CST, 1:200), at 4 °C overnight. The next day, sections were incubated with an HRP-labeled polymer anti-rabbit/mouse secondary antibody for 30 min at RT using a GTVision™ III kit (Cat#: GK500705, Gene Tech). For the control experiments, the primary antibody was replaced by PBS. Finally, positive staining was detected using a 3,3N-diaminobenzidine tetrahydrochloride (DAB) kit and images were analyzed using Image-Pro Plus software (version 6.0).

### Clinical study

A total of 39 patients from the Second Xiangya Hospital of Central South University were enrolled in our study. The study included 27 patients with DM (DM group) and 12 non-DM controls (CON group). The study was approved by the Ethics Committee of the Second Xiangya Hospital of Central South University and conformed to the 1975 Declaration of Helsinki. Informed consents were gained from all subjects. BMF was measured using an enzyme-linked immunosorbent assay (ELISA). Briefly, fasting blood samples were collected using EDTA-containing tubes and stored at -80 °C. Plasma BMF concentration was measured using a commercial sandwich enzyme immunoassay (Cat#: JL40332, Jianglai Biology, China).

Coronary computed tomography (CT) angioplasty was performed using a Siemens Somatom Definition CT multi-layer spiral scanner (Germany). The width of the detector was 128 × 0.6 mm, and the scan thickness was 1.5 mm simultaneously over 50-70 images of fault scans [17]. The CACs was calculated according to the Agatston scoring algorithm, which is the sum of calcium scores of the calcific lesions from the four main branches of the coronary arteries: the left main, left anterior descending, left circumflex and right coronary artery. The area of the calcification lesions multiplied by a fixed coefficient (the maximum pixel density decision) and the total score of the calcification of all faults was defined as the CACs, which was analyzed using Siemens CaScoring software (syngo. via, Siemens Healthcare GmbH) [18].

### Statistical analysis

SPSS (version 26.0) and ﻿GraphPad Prism (version 8.0) were used for data analysis. Quantitative variables were expressed as mean ± SD, or median with minimum and maximum. Qualitative variables were expressed as numbers and percentages. ﻿First, the Shapiro-Wilk test was used to check the normal distribution of the data. A Student’s t-test was used to compare normally distributed data between two different groups, and a one-way ANOVA was used to compare values between more than two groups. The non-parametric Mann-Whitney U test was used to compare non-parametric datasets (non-normal distribution or n<6) between two groups. Categorical variables were compared using chi-squared tests. ﻿Correlations between plasma concentration of BMF and CACs were analyzed using Spearman’s correlation analysis. *p* < 0.05 was considered statistically significant. All experiments were repeated at least three times and results of representative experiments are shown in the figures.

## RESULTS

### Increased expression of BMF and BMF-AS1 in high glucose-induced VSMC calcification and senescence

According to previous studies by us and other scholars, 30 mM of glucose can induce VSMC calcification and senescence [6, 7, 19, 20]. In the present study, glucose-induced BMF-AS1 and BMF expression increased in a concentration- and time-dependent manner, and most significantly on incubation with 30 mM of glucose for 72 h (Supplementary Fig. 3). Additionally, the calcification marker Runx2 and senescence marker p21 increased in VSMCs in a time-dependent manner with 30 mM of glucose incubation (Supplementary Fig. 4). Thus, 30 mM of glucose was used to induce VSMC calcification and senescence in subsequent experiments.

**Table 1:** Comparison of clinical characteristics and plasma BMF concentrations between patients with and without DM.

	DM (n = 27)	CON (n = 12)
Age, years	75.26 ± 9.58	70.92 ± 7.54
M/F, n	14/13	4/8
Current smoking, n (%)	9 (33.3) *	1 (8.3)
Hypertension history, n (%)	22 (88.0) *	5 (41.7)
Coronary heart disease history, n (%)	15 (55.6)	5 (41.7)
BMI, kg/m^2^	23.44 ± 2.65	22.58 ± 3.24
Systolic pressure, mmHg	137.63 ± 15.53	129.83 ± 23.06
Diastolic pressure, mmHg	76.04 ± 10.07	70.58 ± 9.11
TC, mmol/L	3.99 ± 0.86	4.13 ± 0.76
TG, mmol/L	1.29 ± 0.64	1.54 ± 0.54
LDL-C, mmol/L	2.36 ± 0.77	3.12 ± 0.77
HDL-C, mmol/L	1.09 ± 0.21	1.08 ± 0.26
CACs, median (minimum-maximum) BMF, pg/mL	545 (102-1444) * 236.64 ± 24.02 *	62 (0-136) 106.38 ± 6.24

**Figure 1.**
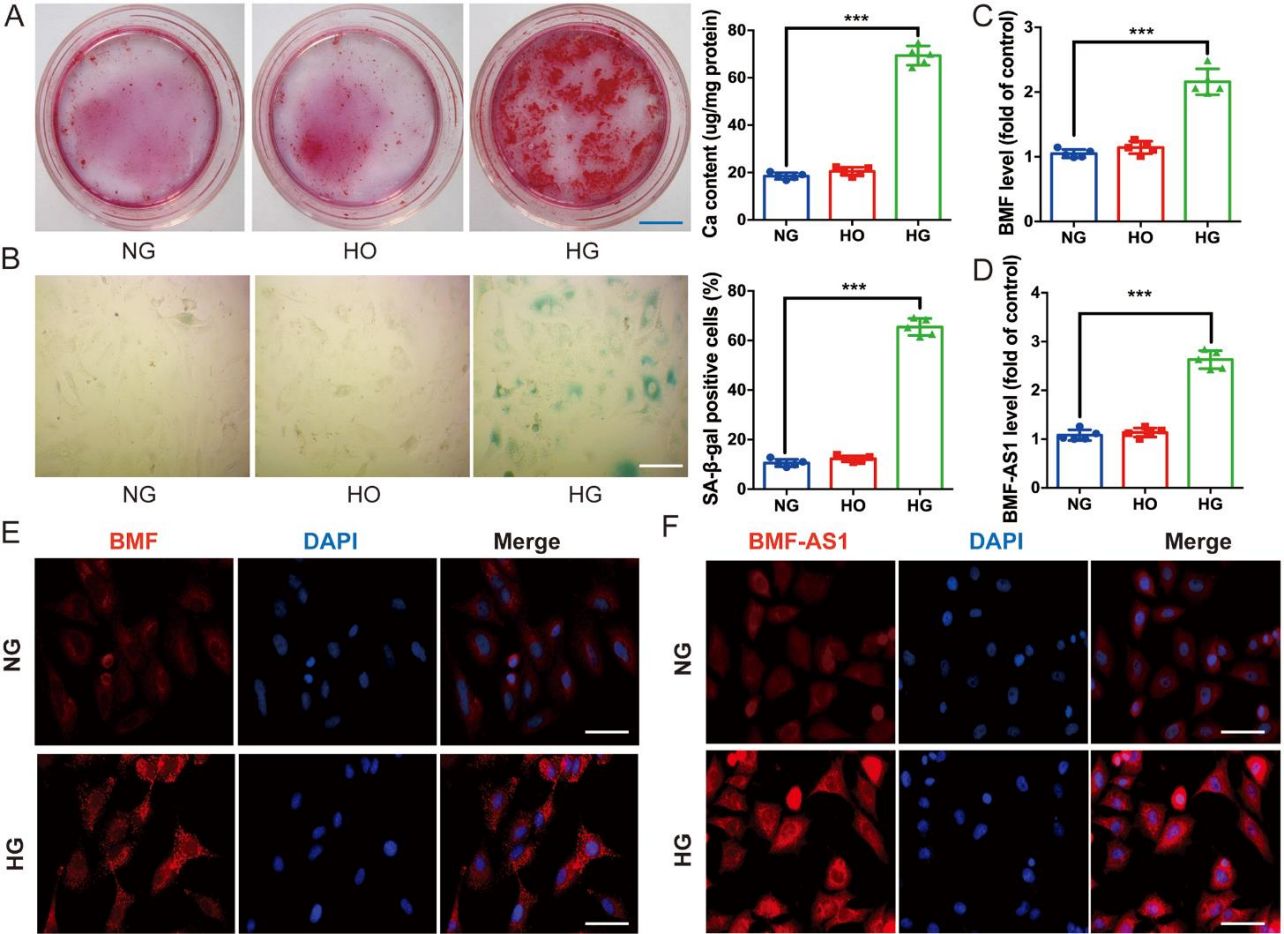
Expression of BMF and BMF-AS1 in high glucose-induced VSMC calcification and senescence. (A) Alizarin Red S staining (n = 5/group). Scale bar ﻿in blue represents 1500 μm. (B) SA-β-gal staining showed senescence in VSMCs treated with NG, HO, or HG for 72 h (n = 5/group). Scale bar ﻿in white represents 100 μm. (C and D) qRT-PCR showing the expression of BMF and BMF-AS1 as fold changes relative to NG group (n = 5/group). (E) Immunofluorescence microscopy of the intracellular location of BMF in VSMCs cultured with NG and HG, respectively (n = 3/group). Scale bar ﻿in white represents 100 μm. (F) FISH showed the presence of BMF-AS1 in both the cytoplasm and nucleus in VSMCs cultured with NG and HG (n = 3/group). Scale bar ﻿in white represents 100 μm. The data were expressed as mean ± SD, one-way ANOVA for Fig 1. A-D. ***p<0.0005, compared with NG group. NG: 5 mM β-GP + 5 mM glucose; HO: 5 mM β-GP + 30 mM mannitol; HG: 5 mM β-GP + 30 mM glucose.

Alizarin Red S staining showed that mineralized nodules were greatly increased (Fig. 1A); and SA-β-gal staining verified that SA-β-gal positive cells were also significantly increased in HG-induced VSMCs (Fig. 1B). However, the high osmolarity control (HO) had a similar effect to NG in terms of the calcification and senescence of VSMCs (Fig. 1A and B). qRT-PCR confirmed that the protein-coding gene BMF, as well as a cognate lncRNA gene BMF-AS1, displayed significantly higher levels in VSMCs treated with HG (Fig. 1C and D). Nevertheless, HO had no significant effect on expression of either BMF or BMF-AS1 compared with NG. Moreover, immuno-fluorescent confocal microscopy demonstrated that BMF was present primarily in the cytoplasm of cultured VSMCs, and HG treatment increased the expression of BMF (Fig. 1E). FISH showed that BMF-AS1 was mainly present in the cytoplasm and at a low level in the nucleus of cultured VSMCs, and HG treatment increased the expression of BMF-AS1 (Fig. 1F).

### BMF-AS1 upregulates BMF expression

**Figure 2.**
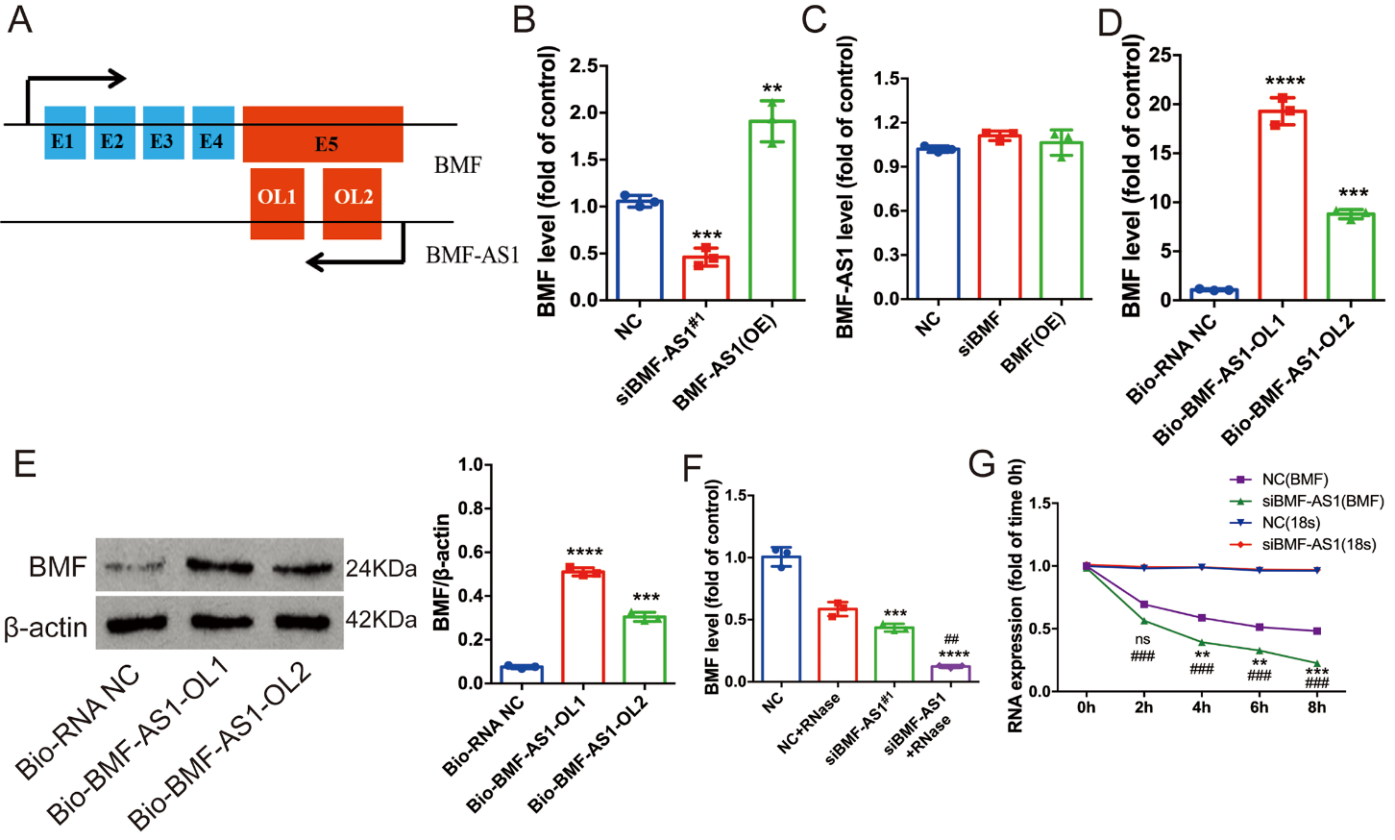
BMF-AS1 up-regulates BMF expression. (A) The relationship between BMF and BMF-AS1. Black arrows indicate transcription direction and blue blocks are exons. The red block in the BMF-AS1 transcript represents the overlapping (OL) region. The red block in BMF represents the fifth exon and also the OL region. “E” followed by a number denotes exon and serial number. (B) BMF mRNA level as fold changes relative to NC group (n = 3/group). (C) BMF-AS1 RNA level as fold changes relative to NC group (n = 3/group). (D and E) Quantification of BMF mRNA level was shown as fold changes relative to Bio-RNA-NC group. The intensity of each band in WB was quantified by densitometry, and data were normalized to the β-actin signal (n = 3/group). (F) Quantification of BMF RNA level shown as fold changes relative to NC group (n = 3). (G) The level of BMF mRNA was measured by qRT-PCR at 0, 2, 4, 6 and 8 h, respectively. 18s-RNA was used as the reference control (n = 3/group). The data were expressed as mean ± SD, one-way ANOVA for Fig. 2B-F. one-way ANOVA and Student’s t-test for Fig. 2G. ****p < 0.0001, ***p < 0.0005, **p < 0.001, compared with NC. ^##^*p* < 0.001, compared with siBMF-AS1 in Fig. 2F. ^###^*p* < 0.0005, compared with NC at the same time point in Fig. 2G. NC: negative control. BMF-AS1 (OE): BMF-AS1 overexpression; BMF (OE): BMF overexpression.

From the UCSC Genome Browser, BMF-AS1 (ENST00000559022 in the Ensemble database) was identified as a single antisense RNA transcribed from the negative strand of the BMF locus. It is located at chromosomal band 15 and consists of two exons. The whole sequence of BMF-AS1 is completely reverse complementary to the fifth exon of BMF, and the two exons of BMF-AS1 are respectively reverse complementary to part of the sequence of the fifth exon of BMF (Fig. 2A, Supplementary. Tab. 4). Since some other lncRNAs have been reported to modulate the expression of nearby protein-coding genes [8, 9], we wondered whether the lncRNA BMF-AS1 would affect the expression of BMF.

First, we transfected VSMCs with a BMF-AS1 vector to overexpress BMF-AS1 or siRNA-BMF-AS1 to knock down the expression of BMF-AS1. We found that the siRNA-BMF-AS1^#1^ variant most effectively inhibited expression of BMF-AS1 (Supplementary Fig. 5A). Therefore, we chose it for the downstream study. qRT-PCR showed that BMF-AS1 overexpression increased BMF mRNA, while BMF-AS1 knockdown in VSMCs decreased BMF levels (Fig. 2B). However, overexpression or knocked down expression of BMF had no significant effect on the RNA level of BMF-AS1 in VSMCs (Fig. 2C). The effect of siRNA-BMF was verified by qRT-PCR (Supplementary Fig. 5B). Secondly, the RNA pull-down assay showed that biotin-labeled BMF-AS1 increased the level of both BMF mRNA and protein, and the overlapping 1 region of BMF-AS1 (BMF-ASl-OL1) bound with BMF more prominently (Fig. 2D and E). Finally, the RPA demonstrated that BMF-AS1 protects BMF mRNA and knocked down BMF-AS1 accelerates BMF mRNA degradation induced by 5 μg/ml of actinomycin D in cultured VSMCs (Fig. 2F and G).

### BMF-AS1 works together with BMF to promote the calcification and senescence of VSMCs

**Figure 3.**
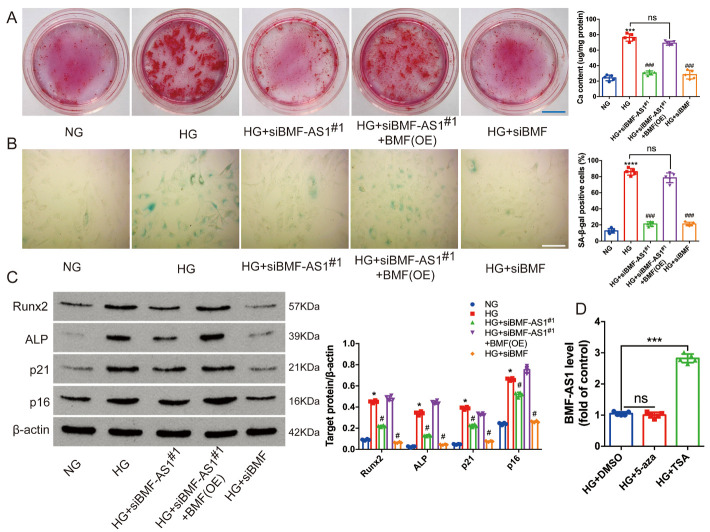
BMF-AS1 together with BMF promotes calcification and senescence *in vitro*. (A) Alizarin Red S staining (n = 5/group). Scale bar ﻿in blue represents 1500 μm. (B) Quantification of SA-β-gal-stained positive cells is shown. Scale bar ﻿in white represents 100 μm (n = 5/group). (C) Representative images of WB analyses of Runx2, ALP, p21 and p16 in VSMCs with different treatments. The intensity of each band in WB was quantified by densitometry, and data were normalized to the β-actin signal (n = 5/group). (D) BMF-AS1 RNA level shown as fold changes relative to HG+DMSO group (n = 3/group). The data were expressed as mean ± SD, one-way ANOVA for Fig. 3A-D. ****p < 0.0001, ***p < 0.0005, *p < 0.05, compared with NG. ^###^*p* < 0.0005, ^#^*p* < 0.05, compared with HG. ns: non-significant (Fig. 3A-C). ***p < 0.0005, compared with HG+DMSO (Fig. 3D). NG: 5 mM β-glycerophosphate + 5 mM glucose; HG: 5 mM β-glycerophosphate + 30 mM glucose; BMF (OE): BMF overexpression. DMSO: dimethylsulfoxide;

To evaluate the role of BMF-AS1 and BMF in the calcification and senescence of VSMCs, their expression was knocked down using siBMF-AS1^#1^ and siBMF, respectively. The results showed that knocking down BMF-AS1 or BMF markedly attenuated VSMC calcification and senescence induced by high glucose, confirmed by a decrease in Runx2, ALP, p21 and p16 expression, mineralized nodules, and SA-β-gal staining positive cells (Fig. 3A-C). Interestingly, the inhibitory effect of BMF-AS1 deficiency on VSMC calcification and senescence was greatly reduced by BMF overexpression, as indicated by an increase in Runx2, ALP, p21 and p16 expression, mineralized nodules, and SA-β-gal positive cells (Fig. 3A-C).

In contrast, VSMC calcification and senescence were greatly increased when BMF-AS1 or BMF were overexpressed. Moreover, VSMC calcification and senescence were almost abolished by knockdown of BMF using siBMF, which was confirmed by Alizarin Red S staining, SA-β-gal staining and Runx2 and p21 expression (Supplementary Fig. 6). In addition, treating VSMCs with the histone deacetylase inhibitor trichostatin A (TSA) greatly increased the expression of BMF-AS1, whereas 5-aza-2’-deoxycytidine, a cytidine analogue and methyltransferase inhibitor, did not (Fig. 3D).

### Bmf^-/-^ inhibits artery calcification and aging in diabetic mice

**Figure 4.**
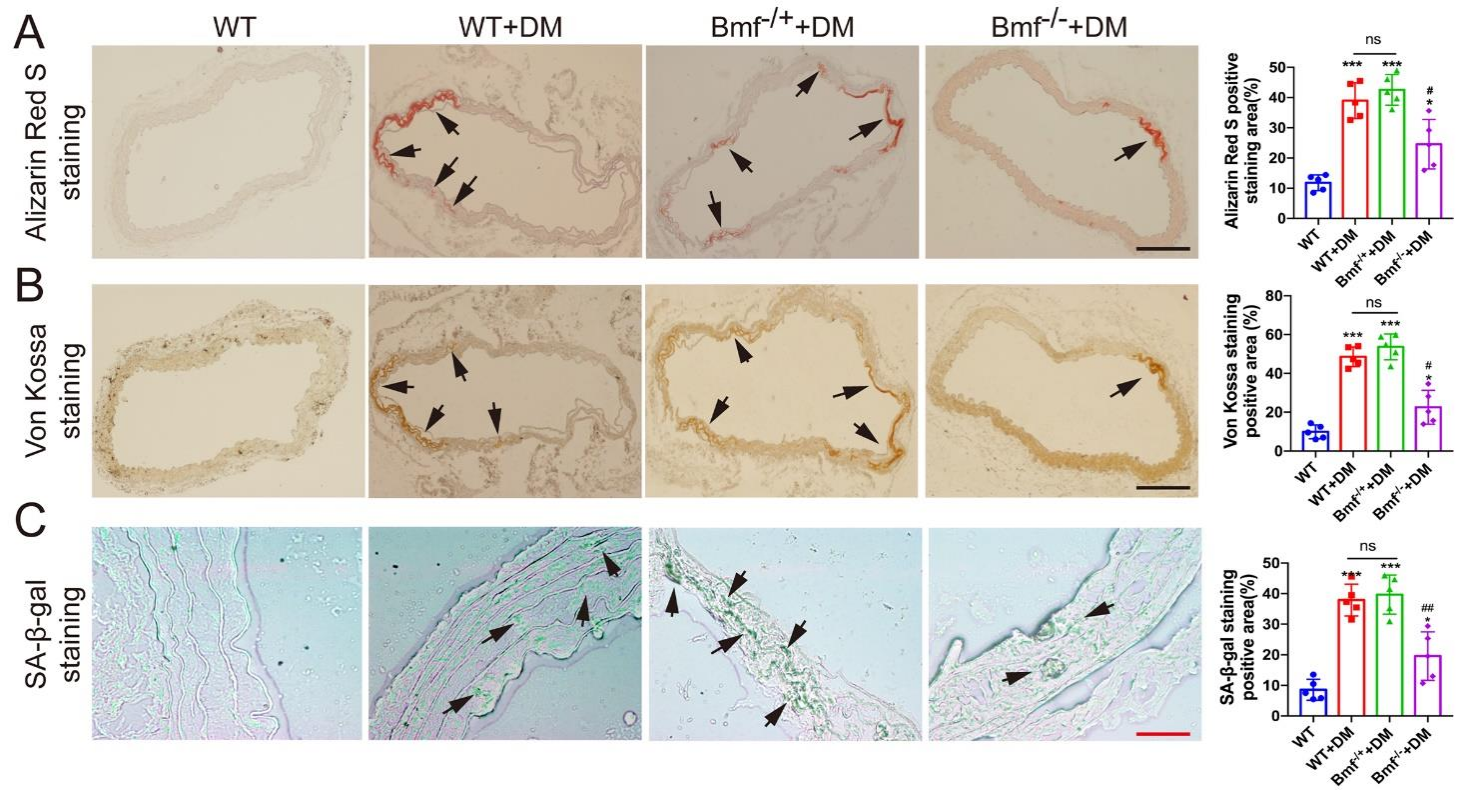
Bmf^-/-^ inhibits artery calcification and aging in diabetic mice. (A and B) Alizarin Red S staining and Von Kossa staining showing calcified aorta from WT, WT diabetic mice, Bmf^-/+^ diabetic mice and Bmf^-/-^ diabetic mice (n = 5/group). Black arrows indicate calcified vessels. Scale bar ﻿in black represents 200 μm. (C) SA-β-gal staining showing aged aortas from the four groups of mice (n = 5/group). Green staining indicates aged tissues and black arrows indicate aging vessels. Scale bar ﻿in red represents 50 μm. The data were expressed as mean ± SD, one-way ANOVA for Fig 4. A-C. Quantification of positively stained areas was measured by Image-Pro Plus software (version 6.0). ***p < 0.0005, *p < 0.05, compared with WT.^##^*p* < 0.001, ^#^*p* < 0.05, compared with Bmf^-/+^ diabetic mice. ns: non-significant. WT: wild type; DM: diabetes mellitus.

**Figure 5.**
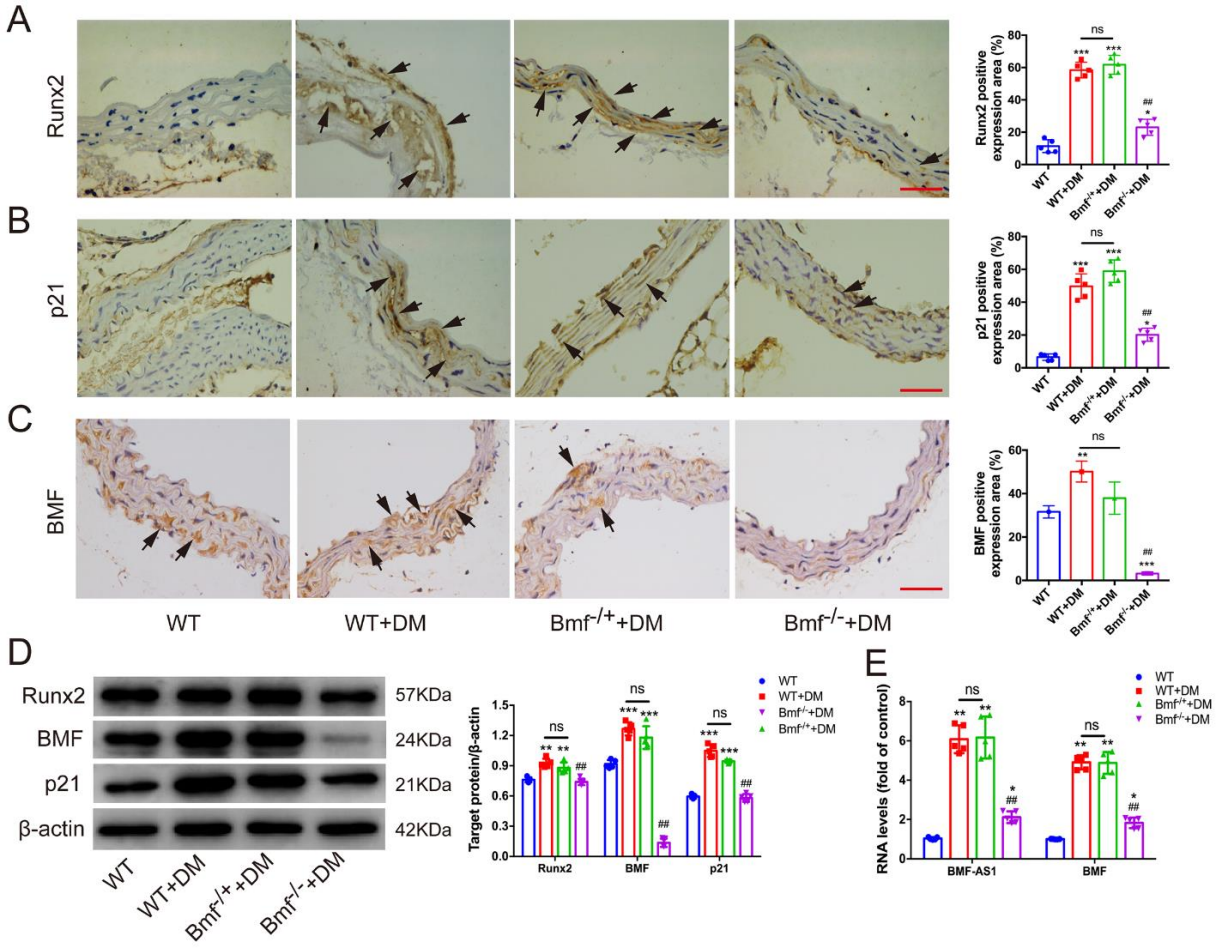
Runx2 and p21 are decreased in Bmf^-/-^ diabetic mice. (A-C) The expression of Runx2 (A), p21 (B) and BMF (C) in aorta tissues from the four groups of mice were examined by immunohistochemistry (IHC). Black arrows indicate the positive expression of Runx2, p21 and BMF (n = 5/group). Scale bar ﻿in red represents 50 μm. (D) The protein level of Runx2, BMF and p21 in aorta tissues from the four groups of mice. The intensity of each band in WB was quantified by densitometry, and data were normalized to the β-actin signal (n = 5/group). (E) ﻿The expression of BMF-AS1 and BMF in aorta tissues as fold changes relative to WT group (n = 5/group). The data were expressed as mean ± SD, one-way ANOVA for Fig. 5A-E. Quantification of positively stained areas was measured by Image-Pro Plus software (version 6.0). ***p < 0.0005, **p < 0.001, *p < 0.05, compared with WT.^##^*p* < 0.001, compared with Bmf^-/+^ diabetic mice. ns: non-significant. WT: wild type; DM: diabetes mellitus.

Finally, Bmf^-/-^ diabetic mice were assessed to evaluate the effect of Bmf on artery calcification and aging *in vivo*. Alizarin Red S and Von Kossa staining showed that artery calcification was significantly attenuated in Bmf^-/-^ diabetic mice compared with WT diabetic mice and Bmf^-/+^ diabetic mice (Fig. 4A and B). Meanwhile, SA-β-gal staining showed that artery aging was also greatly improved in Bmf^-/-^ diabetic mice (Fig. 4C). However, there was no significant difference between WT diabetic mice and Bmf^-/+^ diabetic mice (Fig. 4A-C). Moreover, the expression of both Runx2 and p21 was significantly lower in Bmf^-/-^ diabetic mice, confirmed by immunohistochemistry (Fig. 5A and B). WB analysis also demonstrated that the levels of both Runx2 and p21 proteins were significantly lower in Bmf^-/-^ diabetic mice (Fig. 5D). Additionally, the level of both BMF mRNA and protein were markedly lower in arteries derived from Bmf^-/-^ diabetic mice (Fig. 5C-E). Furthermore, qRT-PCR showed that the expression of BMF-AS1 was also remarkably lower in Bmf^-/-^ diabetic mice (Fig. 5E).

### Plasma BMF concentration is increased in patients with DM and is positively correlated with CACs

**Figure 6.**
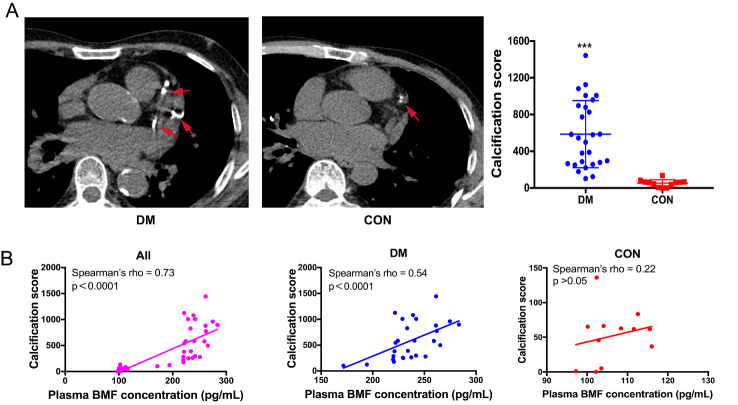
Plasma BMF concentration is positively correlated with CACs. (A) Coronary CT angioplasty of artery calcification (n=27 in DM group, n=12 in CON group). (B) Correlation analysis showed that plasma BMF concentration is positively correlated with CACs (n=39 in all subjects, n=27 in DM group, n=12 in CON group). Red arrows indicate calcification lesions in the coronary artery. Non-parametric Mann-Whitney U test for Fig. 6A. ﻿Correlations between plasma BMF concentration and CACs were analyzed using a Spearman’s correlation analysis (p-value and ﻿Spearman’s rho were indicated ﻿on top of each graph. n=27 in DM group, n=12 in CON group). ***p < 0.0005, compared to CON group. DM: diabetes mellitus; CON: non-DM controls. CACs: coronary artery calcification score.

To further investigate the role of BMF in diabetic vascular calcification, the plasma BMF concentration and CACs in patients with DM were measured. The DM group had significantly higher prevalence rates of smoking and hypertension, while no significant difference was found in terms of age, percentages of coronary heart disease, body mass index, blood pressure or blood lipids between the two groups. Importantly, patients with DM had significantly higher CACs than non-DM controls (median 545 vs 62, *p* < 0.05; Tab. 1, Fig. 6A). The plasma BMF concentration was significantly higher in patients with DM than in CON participants (236.64 ± 24.02 pg/mL vs. 127.55 ± 27.78 pg/mL, *p* < 0.05). ﻿When taking all subjects together, there was a significant positive correlation between CACs and plasma BMF concentration (r = 0.73, *p* < 0.0001). ﻿The correlation was weaker in the CON group (r = 0.22, *p* > 0.05) and stronger in the DM group (r = 0.54, *p* < 0.0001; Fig. 6B).

## DISCUSSION

One of the key findings of the present study is that we described an uncharacterized lncRNA, BMF antisense RNA 1 (BMF-AS1), and its cognate gene, BMF. We found that both VSMCs induced by high glucose and aorta tissues from diabetic mice showed increased expression of the BMF and BMF-AS1. Our study suggested that BMF-AS1 physically interacts with BMF and up-regulates it at the mRNA and protein levels in VSMCs. Additionally, we found that BMF-AS1 and BMF promote VSMC calcification and senescence; and knockdown of BMF-AS1 and BMF attenuates the calcification and senescence of VSMCs, whereas overexpression of that aggravates it. *In vivo*, knocking out the expression of Bmf (Bmf^-/-^) reduces vascular calcification and aging in diabetic mice. Moreover, increased plasma BMF concentration is found in patients with DM and is positively correlated with CACs.

DM is associated with greatly accelerated rates of micro- and macrovascular complications, leading to a significant increase in morbidity and mortality [21, 22]. The key process in vascular calcification is the trans-differentiation of VSMCs into osteoblast-like cells, accompanied by increased expression of osteoblast-related genes (e.g., ALP, Runx2) and mineral nodule formation, resulting in media calcification [2, 23]. In addition, the phenotypic changes of aging are governed by specific alterations in some aging-related proteins, such as p16 and p21, as well as in SA-β-gal staining [7, 24]. Although high glucose is the main reason behind vascular calcification and aging in patients with DM [25, 26], a previous study showed that long-term intensive hypoglycemia control did not have a macrovascular benefit and did not reduce the risk of death or macrovascular complications [27]. Therefore, experts have emphasized that the clinical benefits of vascular protection by controlling hypoglycemia alone are limited, and that new approaches are needed to protect against vascular calcification and aging in patients with DM.

LncRNAs are known to be of great importance in diverse biological processes, including cell proliferation, differentiation, metabolism, genomic regulation, and the immune response [28]. There is increasing evidence that lncRNAs are important modulators in DM and its related complications [10, 22]. For example, Das et al. demonstrated that the lncRNA Dnm3os mediates novel mechanisms for increased macrophage inflammatory gene expression in DM [10]. Interestingly, studies have found that some lncRNAs, classified as antisense transcripts, are defined as RNAs that are reverse complements of their endogenous sense counterparts [29, 30]. Antisense transcripts also play an important role in regulating adjacent coding genes and are involved in suppression, activation, and homeostatic adjustment [8, 31]. Hu et al. reported that a lncRNA, nexilin F-actin binding protein antisense RNA 1 (NEXN-AS1), modulates the expression of the actin-binding protein NEXN and plays a protective role against atherosclerosis [8]. Only a few studies have reported the role of antisense RNAs in DM and its complications [32, 33]. For instance, the lncRNA ArfGAP with RhoGAP domain, ankyrinrepeat and PH domain1 antisense RNA2 (ARAP1-AS2) promotes high glucose-induced renal proximal tubular cell injury by interacting with ARAP1 [32]; and sperm-associated antigen 5 (SPAG5-AS1) inhibits autophagy and aggravates apoptosis of podocytes via the SPAG5/AKT/mTOR pathway in diabetic nephropathy [33]. Accordingly, the present study identified a previously uncharacterized lncRNA, BMF-AS1, and its cognate gene, BMF. The data demonstrated that BMF-AS1 interacts with BMF and upregulates it at both the mRNA and protein levels. Moreover, knocking down BMF-AS1 and BMF attenuates calcification and senescence in VSMCs; and the *in vivo* study showed that Bmf deficiency alleviates vascular calcification and aging in diabetic mice.

BMF, a pro-apoptotic gene, has been found to play a key role in regulating different cell functions under hyperglycemia [11, 12]. It has been demonstrated that miR-29b binds directly to the 3’-UTRs of Bmf and downregulates Bmf expression, which results in an increase in osteoclast survival, but not in osteoclast differentiation [34]. Additionally, ﻿Koike et al. demonstrated that advanced glycation end-products induce apoptosis in VSMCs, leading to their calcification [35]. In addition, Garnet et al. reported an increased expression of Bmf in the renal proximal tubular cells of diabetic mice, which in turn promotes cell apoptosis [11]. Moreover, our previous study demonstrated for the first time that BMF promotes VSMC calcification and senescence induced by high glucose [6]. However, the role of BMF in the calcification and aging of arteries *in vivo* has not been defined. In the present study, we found increased expression of Bmf in the arteries of diabetic mice, while knockout of Bmf attenuated diabetic artery calcification and aging. These results suggest that Bmf is associated with diabetes-induced calcification and aging of arteries *in vivo*. However, BMF-AS1 was lower in Bmf^-/-^ mice, which was different from the results seen *in vitro*. Since the *in vivo* environment is more complex than that *in vitro*, the specific mechanisms remain to be further explored in the future.

Coronary artery calcification (CAC) is an important form of vascular calcification, and the CACs detected by electron beam CT is often used to evaluate the severity of arterial calcification [36]. The quantity of CAC (determined by CT) correlates directly with the quantity of coronary atherosclerotic plaque in necropsy studies [18, 37]; and patients with DM had a higher CACs than those without DM [38]. Additionally, CACs can predict mortality from cardiovascular diseases and can be used as a risk stratification tool for determining cardiovascular risk in patients with T2DM [39, 40]. In this study, we observed a significantly higher CACs and increased plasma BMF concentration in patients with DM, as well as a positive correlation between CACs and plasma BMF concentration. These data suggest that plasma BMF concentration may be used as a biomarker to evaluate arterial calcification in patients with DM.

It is well known that epigenetic modifications, especially histone methylation and acetylation, play important roles in tissue-specific gene expression, and lncRNAs may be regulated in a similar manner to protein-coding genes [41, 42]. For example, H3K9Ac acetylation involving activation of the promoter partially contributes to the up-regulation of Hoxaas3 in pulmonary hypertension [41]. In line with this finding, we observed that the histone deacetylase inhibitor TSA, rather than 5-aza-2’-deoxycytidine, greatly increased the expression of BMF-AS1 in VSMCs. However, the detailed mechanisms modulating BMF-AS1 expression in VSMC calcification and senescence should be explored further.

## Conclusions

In summary, the present study demonstrates the key role of BMF-AS1/BMF in promoting diabetic vascular calcification and aging both *in vitro* and *in vivo*. That is, BMF-AS1 can promote high glucose-induced VSMC calcification and senescence by regulating the actin-binding protein BMF. Additionally, we verified the role of Bmf in artery calcification and aging in diabetic mice using Bmf^-/-^ mice. Moreover, we also observed a more severe CAC accompanied by a higher plasma BMF concentration in patients with DM and found that CACs are positively correlated with plasma BMF concentration. Therefore, this study provides new insights into the diagnosis and treatment of diabetic vascular calcification and aging, which may lead to a decrease in the rate of vascular calcification and aging-related cardiovascular events.

## Supplementary Materials

The Supplementary data can be found online at: www.aginganddisease.org/EN/10.14336/AD.2021.0627.
