# Supplementary material for: BMF-AS1/BMF Promotes Diabetic Vascular Calcification and Aging both In Vitro and In Vivo
Source: Aging Dis. 2023 Feb 1;14(1):170–83. doi: 10.14336/AD.2022.0427 (PMC9937703; doi:10.14336/AD.2022.0427)
Supplement: Supplementary file 1 — The Supplementary data can be found online at: www.aginganddisease.org/EN/10.14336/AD.2021.0627. [file AD-14-1-170-s.pdf]

SUPPLEMENTARY DATA

BMF-AS1/BMF Promotes Diabetic Vascular Calcification and Aging both *In Vitro* and *In Vivo*

Xiao Lin[1,3#], Qun-Yan Xiang[1,2#], Shuang Li[1,2], Wan-Ling Song[4], Yan-Jiao Wang[1,2], Yu-Qing Ni[1,2], Yan Zhao[1,2], Chen Li[1,2], Yi Wang[1,2], Hua-Hua Li[5], Zhen Liang[6], Jun-Kun Zhan[1,2*], You-Shuo Liu[1,2*]

© 2022. Lin X et al. Published online at http://www.aginganddisease.org/EN/10.14336/AD.2022.0427

SUPPLEMENTARY DATA

Supplementary Table 1. The sequences of siRNAs used in the study.

Name	sense（5'-3'）	antisense（5'-3'）
SiRNA-BMF	AAGGUGUCAUGCUGCCUUGTT	CAAGGCAGCAUGACACCUUTT
siRNA-BMF-AS1-84	AGAAGUUGGUCAACAGAUCTT	GAUCUGUUGACCAACUUCUTT
siRNA-BMF-AS1-140	UGAUAUUCAGGGUCAAAACTT	GUUUUGACCCUGAAUAUCATT
siRNA-BMF-AS1-312	AAACAUUCCUCAAGUCUAGTT	CUAGACUUGAGGAAUGUUUTT
Negative control FAM	UUCUCCGAACGUGUCACGUTT	ACGUGACACGUUCGGAGAATT

Supplementary Table 2. Nucleotide sequences of primers used in the study.

Gene	Primer sequence (5' to 3')
BMF	Forward: GGCTGATGTGTCTGTGATGG<br>Reverse: TGGAAGGCTCATAGGTTTGG
BMF-AS1	Forward: CTCAGCAAGCCACAAAGCCT<br>Reverse: AGGTTCTTTCTGGCTGGTTG
β-actin	Forward: CATGTACGTTGCTATCCAGGC<br>Reverse: CTCCTTAATGTCACGCACGAT

Supplementary Table 3. The identify primer sequences for $BMF^{-/-}$ mice.

Primer	Sequence (5'→3')	Primer type
P1	TGTCAGGTGGAGCGAGAGGAACTT	Forward
P2	GGGGGCCCAGCACAGACG	Reverse
P3	GGACTCCGGCCCATAAGC	Forward
P4	GGCCAGTGCACTAAAAGAAGAAAA	Reverse

Supplementary Table 4. The length and sequences of OL1 and OL2 region.

RNA sequences	Length (bp)	Sequences (5' to 3')
OL1	175	BMF:<br>CCAACAAAACCCAGGAGGTAGCTGGCAGAGTAGCTGTCAGGAAGGAGAAAGGTTCTTTCTG<br>GCTGGTTGTTTTTAATTGGCTTAGTGATCTAAACTGGCCCCTTCCTCCTCTGCCTGGTGAGTT<br>GGCCTAAACATTCCTCAAGTCTAGCCTCAGGAGACCTGCCCCTCCCCCCCG<br>BMF-AS1:<br>CGGGGGGGAGGGGCAGGTCTCCTGAGGCTAGACTTGAGGAATGTTTAGGCCAACTCACCAG<br>GCAGAGGAGGAAGGGGCCAGTTTAGATCACTAAGCCAATTAAAAACAACCAGCCAGAAAG<br>AACCTTTCTCCTTCCTGACAGCTACTCTGCCAGCTACCTCCTGGGTTTTGTTGG
OL2	286	BMF:<br>ACCTGGATCCTGACCTGTAACCAGCTGAAGACGGTGGAGGCTTTGTGGCTTGCTGAGGGTGG<br>GGGTTGGGAGAGGGACTGGAAACCTTCCTCCTCGGGAAAGAAATGCCTGGGAGGAAGGGA<br>AGCCTGATATTCAGGGTCAAAACAGCCCTTCTAATTCACAACCCCAAAGCAGGGGTTTCTAG<br>AAGTTGGTCAACAGATCTGGTGAGGGAAGTCCCCAGGCCAGACGCTGGACTCCTGCAATGA<br>GGGTGGAGGACTCAGCCTGGCCTCTGCCTGGCCTGTTTCAA<br>BMF-AS1:<br>TTGAAACAGGCCAGGCAGAGGCCAGGCTGAGTCCTCCACCCTCATTGCAGGAGTCCAGCGT<br>CTGGCCTGGGGACTTCCCTCACCAGATCTGTTGACCAACTTCTAGAAACCCCTGCTTTGGGG<br>TTGTGAATTAGAAGGGCTGTTTTGACCCTGAATATCAGGCTTCCCTTCCTCCCAGGCATTTCT<br>TTCCCGAGGAGGAAGGTTTCCAGTCCCTCTCCCAACCCCCACCCTCAGCAAGCCACAAAGCC<br>TCCACCGTCTTCAGCTGGTTACAGGTCAGGATCCAGGT

© 2022. Lin X et al. Published online at http://www.aginganddisease.org/EN/10.14336/AD.2022.0427

SUPPLEMENTARY DATA

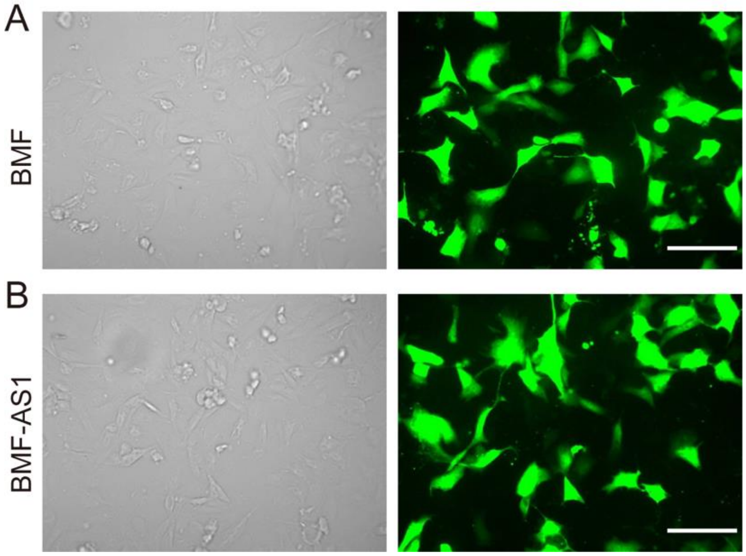

Supplementary Figure 1. The transfection efficacy of BMF-OE and BMF-AS1-OE in VSMCs. The fluorescence image shows the transfection efficiency of the BMF-OE **(A)** and BMF-AS1-OE **(B)** in VSMCs. The green area indicates the positive staining of the BMF-OE (A) or BMF-AS1-OE (B). Representative pictures are shown (n=3/group). Scale bar in white represents 100 μm. OE: overexpression.

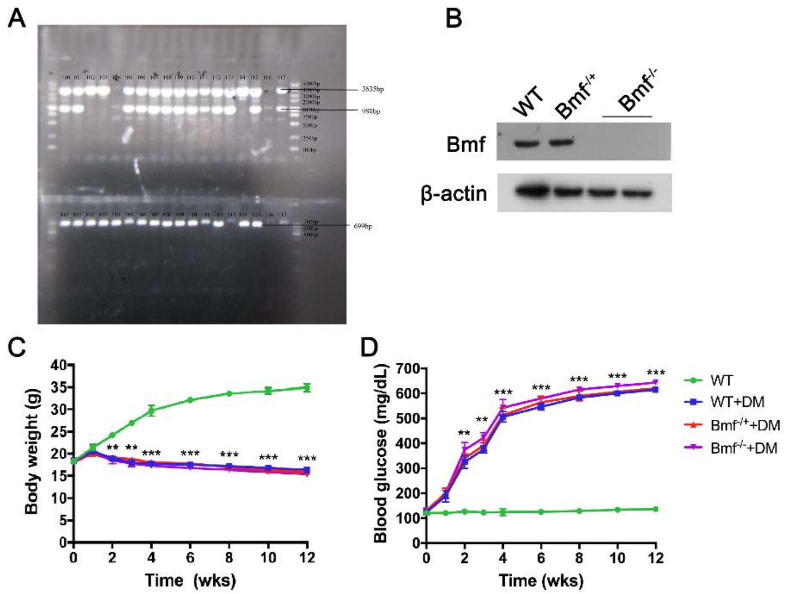

Supplementary Figure 2. The phenotype identification and pathological characters of Bmf$^{-/-}$ diabetic mice. (A) The agarose gel electrophoresis showed that number 104 and 114 were WT mice, number 113 and 117 were homozygous of Bmf knock-out mice (Bmf$^{-/-}$**)**, and the others were heterozygote of Bmf knock-out mice (BMF$^{-/+}$). **(B)** The aorta vessels were isolated from WT, Bmf$^{-/+}$ and Bmf$^{-/-}$ mice, respectively, and the expression of Bmf were measured by WB. The intensity of each band in WB was quantified by densitometry, and data were normalized to the β-actin signal (n=5/group). **(C and D)** The body weight (C) and blood glucose (D) of WT, WT +DM, Bmf$^{-/+}$+DM, Bmf$^{-/-}$+DM mice were measured before and every week after streptozotocin injection. The data were expressed as mean ± SD, one-way ANOVA for Fig. 4A-D. WT: wide type; Bmf$^{-/+}$**:** heterozygote of Bmf knock-out mice; Bmf$^{-/-}$: homozygous of Bmf knock-out mice; DM: diabetes mellitus. ****p*<0.0005, ***p*<0.001, compared with WT.

© 2022. Lin X et al. Published online at http://www.aginganddisease.org/EN/10.14336/AD.2022.0427

SUPPLEMENTARY DATA

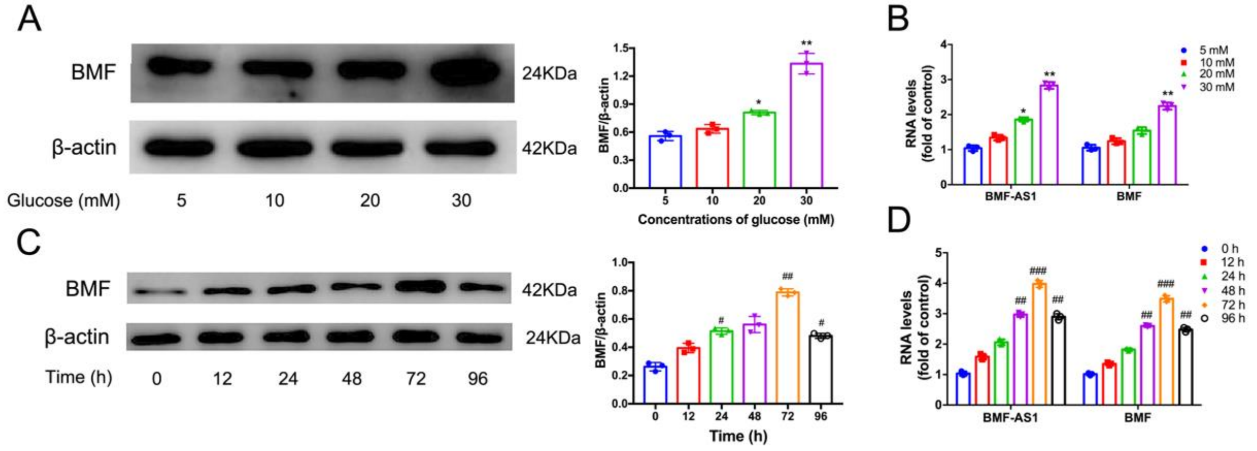

Supplementary Figure 3. The concentration- and time-dependent effects of glucose on BMF and BMF-AS1 in VSMCs. (A and B) VSMCs were incubated with 5, 10, 20, 30 mM glucose for 72 h, respectively. Then WB (A) and qRT-PCR (B) showing the protein level of BMF and RNA level of BMF-AS1 and BMF. The intensity of each band in WB was quantified by densitometry, and data were normalized to the β-actin signal. Quantification of BMF-AS1 and BMF RNA level was shown as fold changes relative to 5mM glucose (n=3/group). **(C and D)** VSMCs were incubated with 30 mM glucose for 0, 12, 24, 48, 72, 96 h, respectively. Then WB (C) and qRT-PCR (D) showing the protein level of BMF and RNA level of BMF-AS1 and BMF. The intensity of each band in WB was quantified by densitometry, and data were normalized to the β-actin signal. Quantification of BMF-AS1 and BMF RNA level was shown as fold changes relative to 0 h (n=3/group). The data were expressed as mean ± SD, one-way ANOVA for Fig. 2A-D. $^{**}p<0.001$, $^{*}p<0.05$, compared with 5mM glucose. $^{\#\#\#}p<0.0005$, $^{\#\#}p<0.001$, $^{\#}p<0.05$, compared with 0 h.

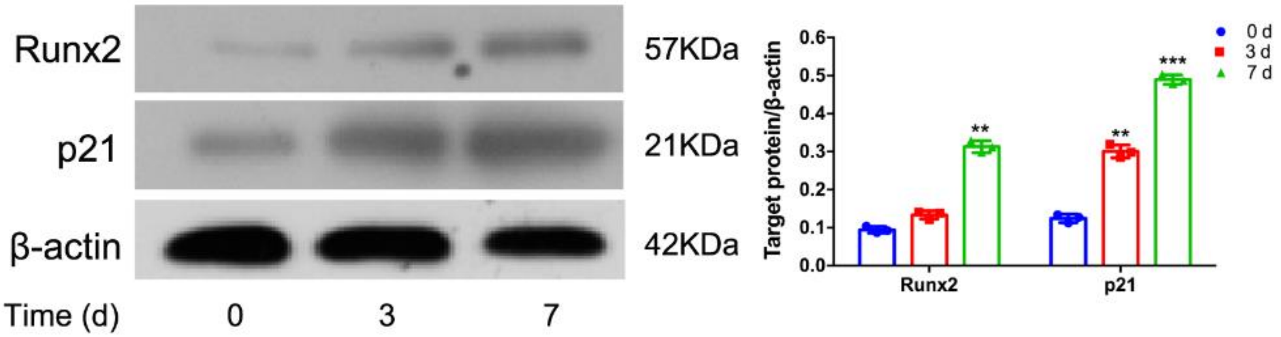

Supplemental Figure 4. The time-dependent effect of HG on VSMC calcification and senescence. VSMCs were incubated with 30 mM glucose for 0, 3, 7 d, respectively. Then WB analysis showing the protein level of Runx2 and p21. The intensity of each band in WB was quantified by densitometry, and data were normalized to the β-actin signal (n=3/group). The data were expressed as mean ± SD, one-way ANOVA for fig. 3. $^{***}p<0.0005$, $^{**}p<0.001$, compared with 0 d. d: day.

© 2022. Lin X et al. Published online at http://www.aginganddisease.org/EN/10.14336/AD.2022.0427

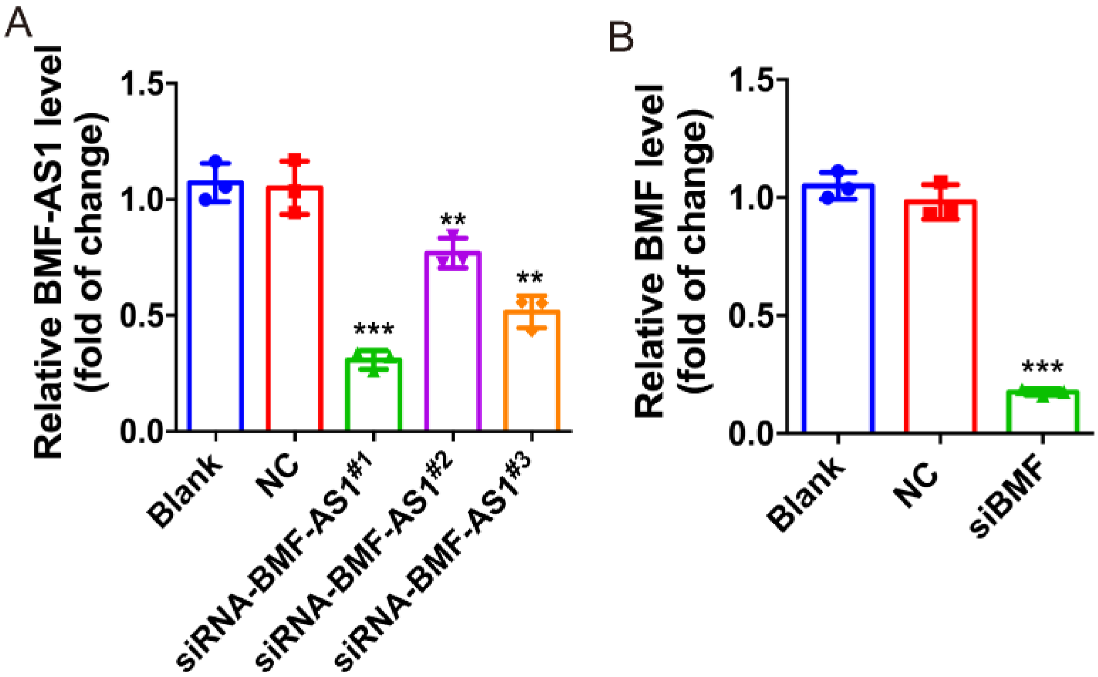

Supplemental Figure 5. The effect of RNA knockdown by siRNA. (A) VSMCs were transfected with three kinds of sequence of siRNA BMF-AS1 and the effects of these siRNA were detected by qRT-PCR. Quantification of BMF-AS1 level was shown as fold changes relative to NC (n=3/group). **(B)** VSMCs were transfected with BMF siRNA and the effect of siBMF were detected by qRT-PCR. Quantification of BMF level was shown as fold changes relative to NC (n=3/group). The data were expressed as mean ± SD, one-way ANOVA for Fig. 5A-B. ***p<0.0005, **p<0.001, Compared with NC. NC: negative control.

© 2022. Lin X et al. Published online at http://www.aginganddisease.org/EN/10.14336/AD.2022.0427

SUPPLEMENTARY DATA

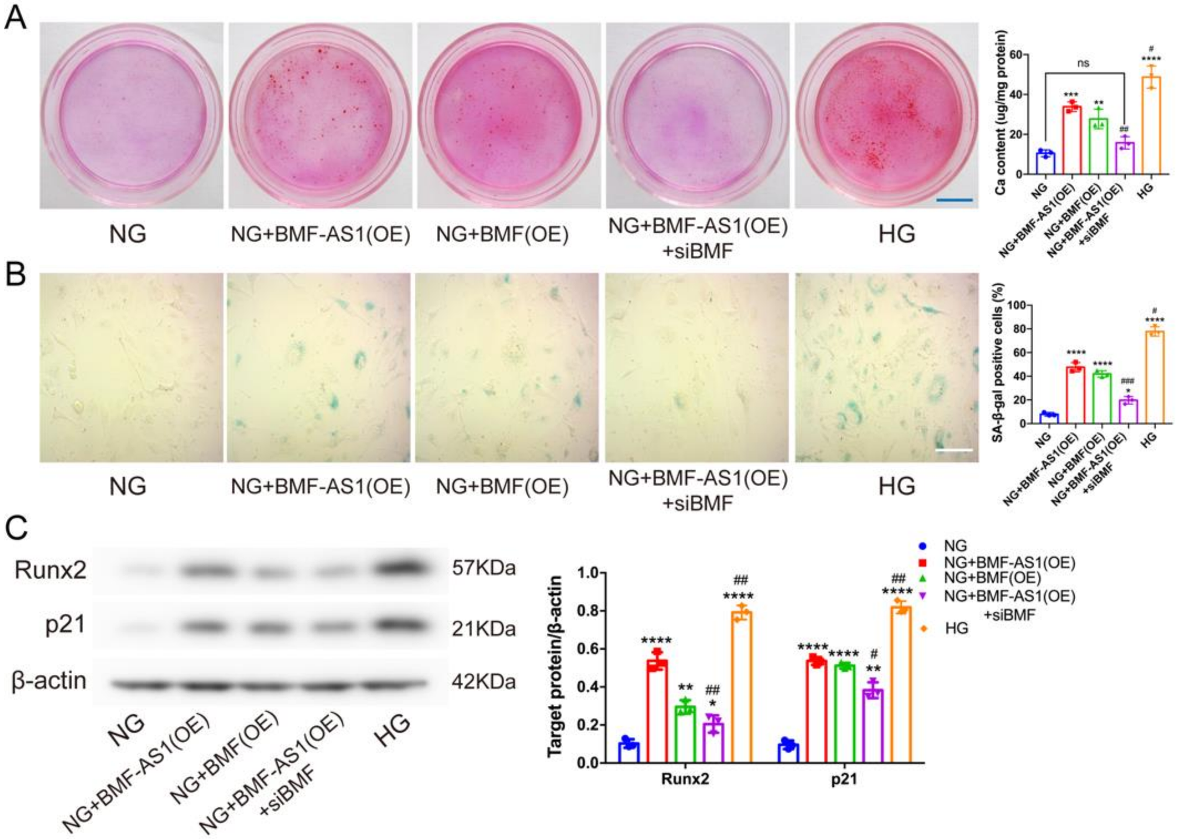

Supplementary Figure 6. BMF-AS1 is dependent on BMF for promoting VSMC calcification and senescence. (A) Alizarin Red S staining detected mineralized nodules in VSMCs with different treatments, and the calcium content was extracted with cetylpyridinium chloride and quantified by spectrophotometry (n=3/group). Scale bar in blue represents 1500 μm. **(B)** SA-β-gal staining detected senescent VSMCs with different treatments, and the quantification of SA-β-gal-stained positive cells is shown (n=3/group). Scale bar in white represents 100 μm. **(C)** Representative images of WB analyses of Runx2 and p21 in VSMCs with different treatment are shown. The intensity of each band in WB was quantified by densitometry, and data were normalized to the β-actin signal (n=3/group). The data were expressed as mean ± SD, one-way ANOVA for Fig. 6A-C. ****p<0.0001, ***p<0.0005, **p<0.001, *p<0.05, compared with NG. $^{\#\#\#}p$<0.0005, $^{\#\#}p$<0.001, $^{\#}p$<0.05, compared with HG+BMF-AS1(OE). ns: no significant. NG: 5 mM glucose; HG: 30 mM glucose.; BMF (OE): BMF overexpression. BMF-AS1(OE): BMF-AS1 overexpression.

© 2022. Lin X et al. Published online at http://www.aginganddisease.org/EN/10.14336/AD.2022.0427
